# Study of inter- and intra-observer reproducibility in the interpretation of [^18^F]choline PET/CT examinations in patients suffering from biochemically recurrent prostate cancer following curative treatment

**DOI:** 10.1186/s13550-014-0025-7

**Published:** 2014-05-30

**Authors:** Clothilde Pegard, Céline Gallazzini-Crépin, Joris Giai, Julien Dubreuil, Cécile Caoduro, Marie-Dominique Desruet, Julie Roux, Alex Calizzano, Daniel Fagret, Chloé Lamesa, Hatem Boulahdour, Jean-Philippe Vuillez

**Affiliations:** 1 Service de Médecine Nucléaire, Centre Hospitalier Universitaire de Besançon (CHU Minjoz), 3 Boulevard Alexandre Fleming, Besançon, 25030, France; 2 Service de Médecine Nucléaire, Centre Hospitalier Universitaire Nord Grenoble, Boulevard de la Chantourne, la Tronche, 38700, France; 3 Centre d’Investigation Clinique, Boulevard de la Chantourne BP 217, Grenoble Cedex 9, 38043, France

**Keywords:** 18F, choline positron emission tomography, Computed tomography, Prostate cancer, Biochemical recurrence, Inter- and intra-observer reproducibility

## Abstract

**Background:**

The aim of this study was to investigate the reproducibility of intra- and inter-observer interpretation of [^18^F]choline positron emission tomography/computed tomography examinations in patients suffering from biochemically recurrent prostate cancer following curative treatment.

**Methods:**

A total of 60 patients with biochemical recurrence after curative treatment were included in this bicentric study. The interpretations were based on a systematic analysis of several anatomic regions and all the four nuclear medicine physicians used identical result consoles. The examinations were interpreted with no knowledge of the patients' clinical context. Two months later, a second interpretation of all these examinations was performed using the same method, in random order.

**Results:**

To evaluate local recurrences, when the prostate is in place, the results showed moderate inter- and intra-observer reproducibility: concordance of all 4 physicians has a Fleiss' kappa coefficient of 0.553 with a confidence interval of (0.425 to 0.693). For patients who had had a prostatectomy, there was excellent concordance for the negative examinations. For the lymphatic basin, inter- and intra-observer reproducibility was excellent with a Fleiss' kappa coefficient of 0.892 with a confidence interval of (0.788 to 0.975). The lymphatic sub-group analysis was also good. For the lymphatic groups in the right or left hemi-pelves, all Fleiss' kappa and Cohen's kappa coefficients are varying from 0.760 to 1 with narrow confidence intervals from (0.536 to 0.984) to (1 to 1) in favour of good/excellent inter-observer reproducibility. To evaluate bone metastasis, inter-observer reproducibility was good with a Fleiss' kappa coefficient of 0.703 and a confidence interval of (0.407 to 0.881).

**Conclusion:**

Our study is at time the only one on the reproducibility of interpretation of [^18^F]choline positron emission tomography/computed tomography examinations, which is a key examination for the treatment of patients suffering biochemical recurrence of prostate cancer. Interpretation of the [^18^F]choline positron emission tomography/computed tomography examination is not so useful at prostate level in patients not previously treated with prostatectomy but has a great interest on patients treated by prostatectomy. It showed good concordance in the interpretation of sub-diaphragmatic lymphatic recurrences as well as in bone metastasis.

## Background

Prostate cancer is the most frequent cancer in men in Western Europe and North America and is estimated to cause death of one of ten men suffering from this cancer [1,2]. For patients suffering from prostate cancer, recurrence after radical treatment is common during the first 10 years. The risk of recurrence is 20% to 50% in patients treated by radical prostatectomy, and 30% to 40% in patients treated with external radiotherapy [3,4].

At present, the detection of recurrent prostate cancer in patients who had curative treatment is done by blood assay of prostate specific antigen (PSA) levels [5-7]. There are currently several possible imaging modalities, often complementary, for detecting recurrence of prostate cancers, including pelvic MRI, endorectal ultrasound, bone scintigraphy, thoracic-abdomino-pelvic (TAP) CT-scan and [^18^F]choline positron emission tomography/computed tomography (PET/CT).

Numerous authors studied the sensitivity and specificity of [^18^F]choline PET/CT for the biochemical recurrence of prostate cancers [8-10], but none of them investigated the variability of interpretations of [^18^F]choline PET/CT examinations. The aim of this bicentric retrospective observational study was to evaluate the inter- and intra-observer variability of interpretations of [^18^F]choline PET/CT examinations in patients suffering from biochemical recurrence of prostate cancer following curative treatment.

## Methods

### Patient population

From all patients that had a [^18^F]choline PET/CT examination at our two centres from 2011 to 2013, we retrospectively included 60 patients that met the following inclusion criteria:

 Males with biochemical recurrence of PSA after curative treatment for prostate cancer including total prostatectomy, external radiotherapy or radium treatment. Biochemical recurrence was defined by PSA level over 0.2 ng/ml if initial treatment was prostatectomy, or PSA level over 2 ng/ml if initial treatment was conformational radiotherapy [5,6].

 [^18^F]choline PET/CT examination with an acquisition protocol including at least one dynamic examination and a later full body examination.

The patient data were anonymized (Table 1). All patients provided informed consent for their participation in this study. Our study protocol was approved by the Institutional Review Boards of both centres.

**Table 1:** Patient characteristics

**Characteristics**	**Value**
Age (years)	
Average	71
Min to max	57 to 86
Median	72
Initial PSA level (ng/ml)	
Average	14.77
Min to max	1.5 to 63
Median	9.80
PSA level; at the time of the PET/CT request (ng/ml)	
Average	6.43
Min to max	0.35 to 154
Median	2.73
Gleason score	
≤7	56
>7	4
NA	5
TNM Stage	
Any T2Nx	2
Any T2N0M0	18
Any T2N1M0	1
Any T1c N0 or Nx	15
Any T3N0M0	8
Any T3N1	5
Any T3Nx	3
NA	8
Initial treatment	
Surg. Only	9
Surg. A nd RT	8
Surg. RT and HT	2
RT only	34
Brachytherapy only	5
Time to double PSA (months)	
>12 months	6
= 12 months	8
<12 months	5
<6 months	4
≤3 months	5
NA	32
Velocity (ng/ml/year)	
Average	2.87
Min to max	0.05 to 15.45
Median	2.03

PSA, prostatic specific antigen; TNM, tumor nodes metastasis; RT, radiotherapy; HT, hormone therapy.

### Acquisition protocol

The acquisition protocol for the [^18^F]choline PET/CT examination was identical for all patients, but different equipment were used at the two centres. For 52 patients at one centre, a PET-CT Scanner (Discovery 690, General Electric, Wakacha, IL, USA) was used with three-dimensional ordered subsets expectation maximization (3D-OSEM) reconstructions having 2 iterations and 24 subsets. For eight patients at the other second centre, a Biograph Duo PET/CT Scanner (Siemens AG, Munich, Germany) with 3D-OSEM reconstructions having three iterations and eight subsets. Six to eight bed positions per patient were acquired from the head to the upper third of the thighs. The PET images were corrected for random coincidences, scatter and attenuation using the CT scan data.

The patients had been fasting for 6 h, non-hydrated, and emptied their bladder before the examination. Interpretation was based on an early dynamic image acquisition, done after a bolus injection of [^18^F]choline (activity 5 MBq/kg) under the camera, and on a late image acquisition. The image acquisitions included 10 consecutive 1-min recordings on a single bed centred on the pelvis. Dynamic acquisition started about 2 min after the injection in list mode. An early acquisition centred on the abdomen and pelvis was taken immediately afterwards, from the hepatic dome to beneath the ischia. Immediately after this series of images, the patient was injected with 20 mg of furosemide and was orally hydrated. After 40 min of rest in a chair, a late whole-body acquisition was taken, from the vertex to the mid-thigh.

### Interpretation methodology

The 60 patient examinations were interpreted separately by four nuclear medicine specialists: two senior physicians (CG, CC) and two junior physicians (JD, CP). The senior physicians had at least 3 years of experience in interpreting [^18^F]choline PET/CT results, whereas the junior physicians had 1 year of experience in interpreting [^18^F]choline PET/CT results.

The examinations were interpreted with no knowledge of the patients' clinical context. Each physician made an initial interpretation of the anonymized examinations. Images were interpreted visually based on averaged acquisitions of two time intervals: 1 to 5 and 6 to 10 min. The first interval enabled visualization without distortions caused by bladder activity. The interpretations were based on a systematic analysis of the following anatomic regions: (i) the prostatic bed or the prostate, (ii) the periprostatic lymph node areas, (iii) the perirectal lymph node areas, (iv) medial iliac lymph node areas, (v) lateral iliac lymph node areas and the iliac bifurcation lymph node area, (vi) common iliac lymph node areas, (vii) lumboaortic and mesenteric lymph node areas, (viii) and supra-diaphragmatic lymph nodes and remote metastatic sites: bone, lung, liver, and central nervous system.

Each physician had to visually evaluate the type of foci of increased uptake, with binary rating of ‘0’ for benign/non-significant or ‘1’ for malignant/high-risk, for each anatomical region described above. At least 2 months later, a second interpretation of all pre-existing examinations was performed by each physician separately using the same method, in different order (defined by a random number generator software).

### Statistical analysis

The analyses were performed using the software STATA version 12.0 (College Station, StataCorp LP, TX, USA). The main assessment criterion was the reproducibility of inter- and intra-observer interpretation of [^18^F]choline PET/CT examinations, evaluated by the kappa (κ) statistical test [11]. Cohen's Kappa coefficient (reproducibility between two observers) and the Fleiss' Kappa coefficient (reproducibility between four observers) indicated reproducibility of interpretations: κ > 0.81 is excellent, 0.80 > κ > 0.61 is good, 0.60 > κ > 0.41 is moderate, 0.40 < κ < 0.00 is average, and κ < 0.00 is poor [12]. The kappa coefficients were recorded with their confidence intervals (CI). The null hypothesis was that κ = 0, i.e. no inter- or intra-observer agreement other than pure chance. Moreover, in this study, the *p* values are lower than 5%. For this reason, our results are statistically significant. However, since this study was not a randomized study, the alpha risk is not applicable (as per statistical rules).

The overall conclusion of the PET examination included analysis of the prostate, supra- and sub-diaphragmatic nodes and remote metastatic sites (bone, lung, liver, central nervous system) for a given patient. Statistical analyses were also done independently for each of the aforementioned anatomic regions. Other calculations were made on the pelvic lymphatic nodes of the right and left hemi-pelves. The pelvic lymphatic nodes included all the sub-diaphragmatic lymphatic areas (excluding the inguinal nodes) which were found to be positive or negative by each physician. The right and left hemi-pelves included the common iliac lymph nodes, iliac bifurcation lymph nodes, lateral and medial iliac lymph nodes, periprostatic and obturator lymph nodes on the same side.

## Results

The inter-observer reproducibility for the overall conclusion of the [^18^F]choline PET/CT examination was moderate. Reproducibility between the four physicians had a kappa of 0.499 (CI 0.350 to 0.652). Reproducibility between the two senior physicians had a kappa of 0.454, whereas reproducibility between the two junior physicians had a kappa of 0.537.

When the prostate is in place, the inter-observer reproducibility between all four physicians was moderate with a kappa of 0.553 (CI 0.425 to 0.693). The intra-observer reproducibility was moderate for two physicians and was poor/average for the other two physicians.

For the evaluation of local recurrences for the prostatectomy bed, the kappa coefficient could be calculated but would have no sense. Indeed, only 3 of the 19 operated patients were considered to be positive for the prostatectomy bed. Among these three patients, all four nuclear physicians concluded on recurrence for two patients, and only two nuclear physicians concluded on recurrence for the third patient.

For the pelvic lymph nodes, there was excellent inter-observer reproducibility with a kappa of 0.892 (CI 0.788 to 0.975). For the lymph nodes in the right and left hemi-pelves, there was good to excellent inter-observer reproducibility with a kappa between 0.760 (CI 0.536 to 0.984) and 1 (CI 1 to 1). The left medial iliac lymph nodes had lower inter-observer reproducibility with a kappa of 0.362 (CI −0.008 to 0.662). For some locations, such as the left periprostatic lymph node, the kappa coefficient could not be calculated as there was no positive focus. All the other lymph nodes (lateral iliac lymph nodes iliac bifurcation lymph nodes, common iliac lymph nodes, lumbo-aortic mesenteric lymph nodes) had good reproducibility between the four physicians with kappa between 0.559 (CI −0.021 to 1) and 0.806 (CI 0.587 to 0.965).

For the evaluation of metastatic dissemination, inter-observer reproducibility between the four nuclear physicians was good with a kappa of 0.703 (CI 0.407 to 0.881). For the other secondary locations, lungs and liver, mediastinal lymph nodes, the kappa could not be calculated.

The intra-observer reproducibility of interpretations of the overall conclusion of the [^18^F]choline PET/CT examination was good except for one of the junior physicians.

## Discussion

The precise identification of the site of biochemical recurrence of prostate cancer is important as it enables initiation of curative treatments such as radiotherapy and surgery. These treatments help delay the introduction of hormone therapy, a long-term treatment with only temporary efficacy and with many side effects. In a prospective study of 72 patients, Rigatti et al. [13] showed that salvage lymphadenectomy guided by choline PET for patients suffering biochemical recurrence after curative treatment has raised the prostate cancer remission level by 35% in 5 years. This study shows that progression-free survival is increased by appropriate treatment.

[^18^F]Choline PET/CT has an important position in the diagnosis prior to the treatment of prostate cancers in a situation of biochemical recurrence, considering its high level of sensitivity*.* Giovacchini et al [8] showed a rate of positive diagnosis after prostatectomy of 83% when the PSA level is above 3 ng/ml.

The reproducibility of interpretation of this examination becomes essential when the result leads to a decision on choice of therapy. As far as we know, our study is the first to observe the reproducibility of interpretation of [^18^F]choline PET/CT examinations.

In our study, the inter-observer reproducibility of the PET overall conclusion for [^18^F]choline PET/CT examinations was moderate, and it is particularly lowered by the poor reproducibility of the prostate results.

**Figure 1:**
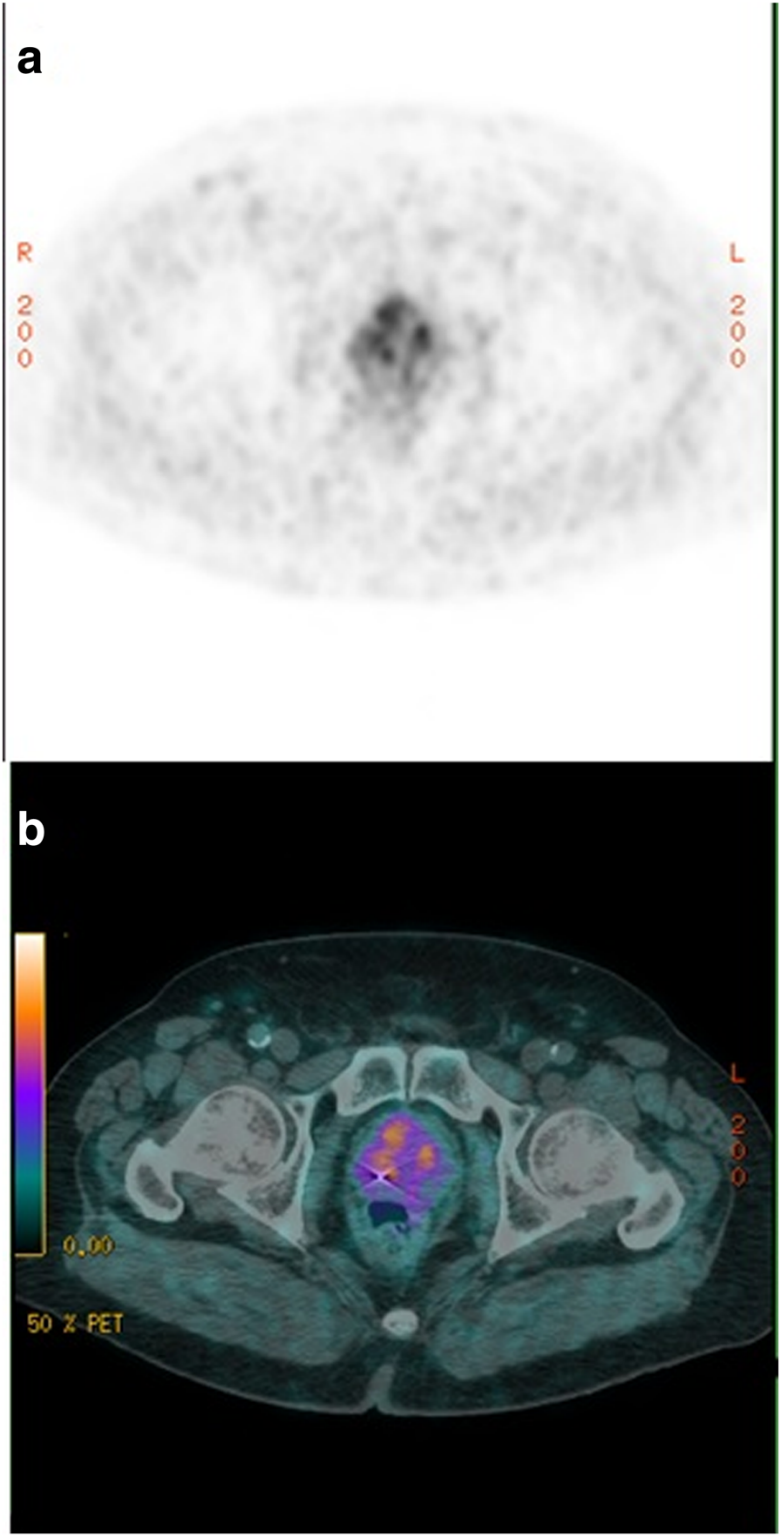
**[**^**18**^F**]choline PET (a) and [**^**18**^**F]choline PET/CT (b) of axial section of pelvis.** This shows a heterogeneity of increased uptake in the prostate. This figure also illustrates a case of discordance between the nuclear medicine physicians (one junior in favor of recurrence and the three other nuclear medicine physicians not in favor of a recurrence).

In the pelvis, evaluation of intraprostatic recurrence is difficult, which was the case in our study (Figure 1). For initial diagnosis, a study showed that [^18^F]choline PET/CT has a high sensitivity in detecting prostate cancer of 86.5% but a lower specificity of 61.9% [14]. Numerous studies showed that differentiating between malignant and benign intraprostatic lesion is difficult using [^18^F]choline PET/CT. Some authors showed many false-positive cases linked with prostatisis [15]. For this reason, it is likely that in our study, the different nuclear physicians interpreted the false-positive results differently because of the low specificity of [^18^F]choline PET/CT when the prostate is in place. Other authors showed an overlap of max standardized uptake values (SUVs) between cancers and benign lesions [16]. This explains why reproducibility is mediocre, as shown by our discordances in interpretation between physicians when the prostate is still in place, which was the case for most of our patients. These results demonstrate the need to define more robust interpretation criteria than those currently in use.

In order to solve this issue, some authors proposed an interpretation method, using the kinetics of choline capture in the prostate. This enables to distinguish a cancer from a benign tumour or prostatitis. Authors showed that choline capture takes place very quickly during the first 30 min and then it remains on a plateau if there is a cancerous lesion. However, this study does not specify whether this has improved sensitivity and specificity [17]. For initial diagnosis, a study showed that MRI (MR imaging +3D MR spectroscopic imaging) has a better sensitivity of prostate cancer than [^18^F]choline PET/CT examination [18]. However, we did not find any studies about the reproducibility of interpretation of prostatic MRI examinations.

**Figure 2:**
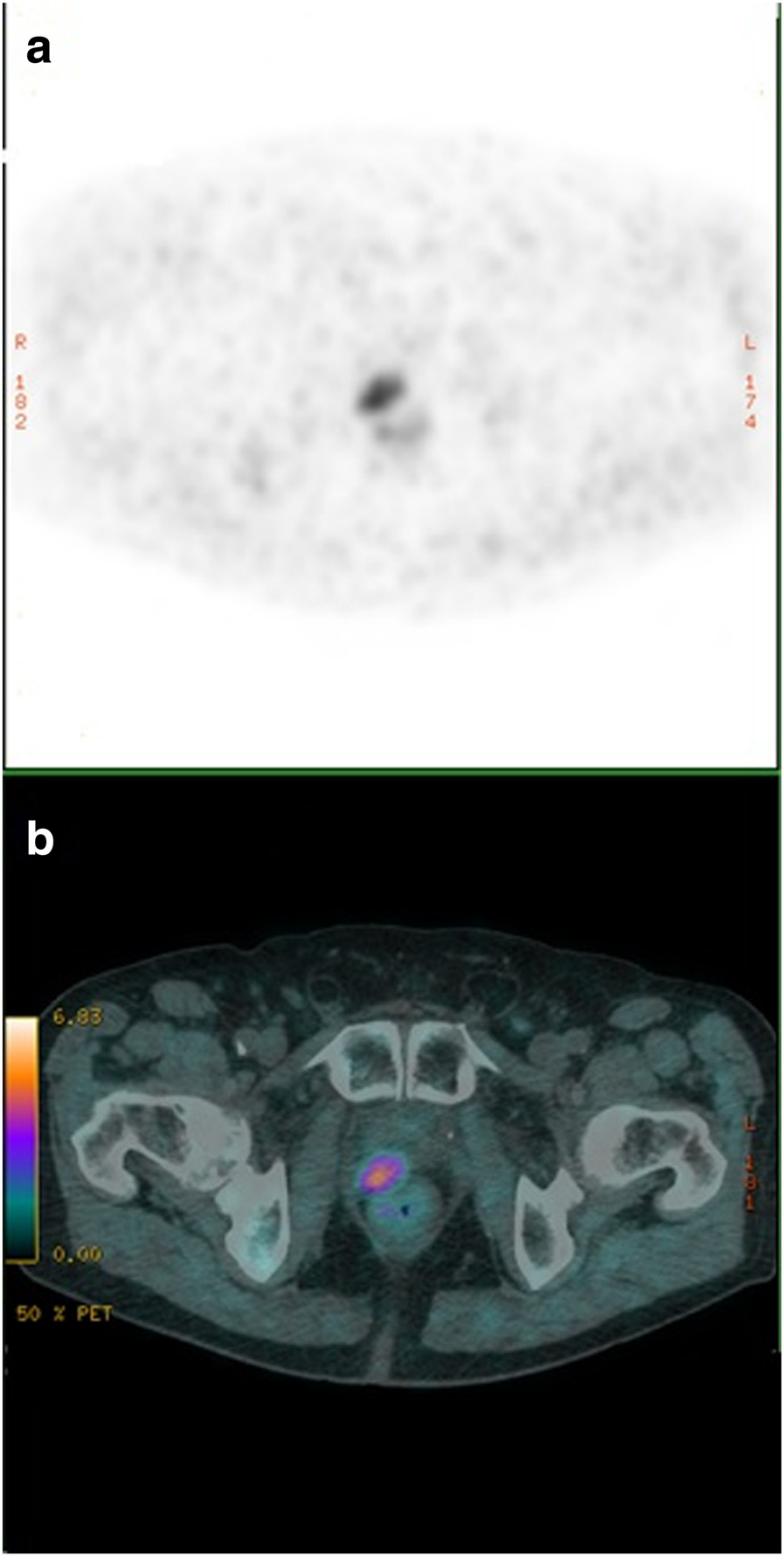
**[**^**18**^F**]choline PET (a) and [**^**18**^**F]choline PET/CT (b) of axial section of pelvis.** This shows a hypermetabolic focus in the prostatectomy bed on the right, suspecting recurrence. The figure also illustrates a case of full concordance between the four nuclear medicine physicians.

In the prostatectomy bed, our results of reproducibility are excellent but concern only 3 positive cases out of 19 patients (Figure 2). Indeed, concordance was 100% for the 16 patients considered to be negative. For the only discordant patient in both inter- and intra-observer status for two physicians, it should be noted that the patient had a total hip arthroplasty which affected the interpretation.

A study shows that [^18^F]choline PET/CT demonstrates sensitivity, specificity, negative predictive value (NPV), and positive predictive value (PPV) of percentages 73%, 88%, 92%, and 61%, respectively, after surgery comparable to magnetic resonance imaging (MRI) at 3 T for a PSA level greater than 1.3 ng/l [19].This study concerned patients who were candidates for salvage radiotherapy with a curative aim, but these studies do not specify reproducibility. In conclusion, reproducibility is excellent for negative cases, which supports the NPV of the examination.

**Figure 3:**
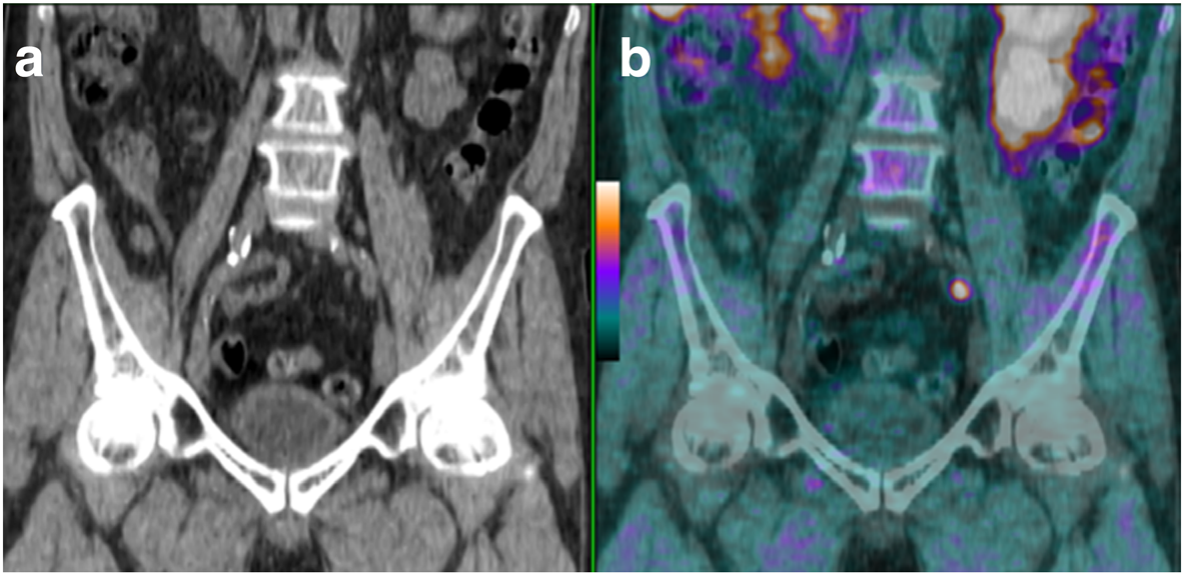
**[**^**18**^F**]choline CT (a) and [**^**18**^**F]choline PET/CT (b) of abdomino-pelvic coronal section.** This shows a hypermetabolic focus projected on an adenopathy of the left iliac bifurcation, suspecting recurrence. This figure also illustrates a case of full concordance between the four nuclear medicine physicians.

In the pelvis lymph nodes, our inter- and intra-observer interpretation reproducibility was excellent (Figure 3). These results are coherent with studies concerning the performance of this examination. Scattoni et al. showed that [^11^C]choline has a good PPV [10]. De Jong et al. showed the [^11^C]choline PET had a sensitivity, specificity, and accuracy of 80%, 96%, and 93%, respectively, for detecting lymphatic metastasis [9]. The nuclear medicine physicians in our study obtained good reproducibility of interpretation in both inter- and intra-observer situations in sub-diaphragmatic lymph nodes. This supports the results of other studies on the contribution of [^18^F]choline PET/CT in detecting abdomino-pelvic lymphatic recurrence in a situation of biochemical recurrence [13]. Another study showed [^18^F]choline PET/CT examination which in a situation of recurrence found a high PPV estimated at 86% and a less sensitivity at 72%, linked to micrometastatic involvement [10].

These results are all the more significant in that reproducibility was good, or even excellent for both seniors and juniors. This leads to the assumption that any nuclear medicine physician with [^18^F]fluorodeoxyglocose (FDG)/PET experience can interpret an [^18^F]choline PET/CT examination just as efficiently, particularly for detecting sub-diaphragmatic lymphatic recurrences [5,6].

These conclusions apply to the various lymphatic areas as shown in Tables 2 and 3. The limits of our study concern several points. The use of kappa coefficients for the inter-observer reproducibility was not possible in some locations, given the low prevalence of the positivity of the expected/researched sign of our sample. Few or no tumour involvement was suspected not only in some locations, such as lung, liver, muscles, and mediastinal and inguinal nodes, but also in medial left iliac lymph nodes. The low number of positive nodes might explain the poor kappa coefficient in this latter location. Some wide confidence intervals are giving us another limit of this study, as is the case for the left and right medial iliac lymph nodes.

**Table 2:** Inter-observer reproducibility of binary interpretations (negative/benign/non-significant or positive/malignant/high-risk)

**Locations**	**Inter-observer agreement at first interpretation**		
	**kappa [confidence interval]**		
	**Two senior physicians**	**Two junior physicians**	**All four nuclear physicians**
Prostate and prostatic bed	0.337 [0.115 to 0.559]	0.661 [0.473 to 0.848]	0.550 [0.425 to 0.692]
Prostate	0.328 [0.096 to 0.561]	0.643 [0.444 to 0.842]	0.553 [0.425 to 0.693]
Prostatectomy bed	NA	NA	NA
Pelvic LN	0.921 [0.812 to 1.000]	0.889 [0.766 to 1.000]	0.892 [0.788 to 0.975]
R hemi-pelvic LN	1.000 [1.000 to 1.000]	0.777 [0.569 to 0.896]	0.833 [0.626 to 0.958]
L hemi-pelvic LN	0.795 [0.571 to 1.000]	0.760 [0.536 to 0.984]	0.819 [0.600 to 0.942]
R periprostatic LN	0.792 [0.396 to 1.000]	1.000 [1.000 to 1.000]	0.885 [−0.004 to 1.000]
L periprostatic LN	0.000 [0.000 to 1.000]	0.000 [0.000 to 1.000]	0.212 [−0.008 to 0.328]
R medial iliac LN	0.550 [0.107 to 0.993]	0.550 [0.107 to 0.993]	0.646 [0.165 to 0.937]
L medial iliac LN	−0.023 [−0.070 to 0.025]	0.487 [−0.113 to 1.000]	0.362 [−0.008 to 0.662]
R lateral iliac LN and bifurcation iliac LN	0.880 [0.648 to 1.000]	0.815 [0.568 to 1.000]	0.774 [0.494 to 0.948]
L lateral iliac LN and bifurcation iliac LN	0.659 [0.036 to 1.000]	0.483 [−0.131 to 1.000]	0.559 [−0.021 to 1.000]
R common iliac LN	0.848 [0.557 to 1.000]	0.677 [0.381 to 0.973]	0.669 [0.269 to 0.891]
L common iliac LN	0.699 [0.379 to 1.000]	0.838 [0.620 to 1.000]	0.806 [0.587 to 0.965]
Inguinal LN	NA	NA	NA
Lumboaortic LN	0.900 [0.707 to 1.000]	0.815 [0.568 to 1.000]	0.888 [0.646 to 1.000]
Mediastinal LN	NA	NA	NA
Bone	0.712 [0.445 to 0.978]	0.608 [0.323 to 0.893]	0.703 [0.407 to 0.881]
PET overall conclusion	0.455 [0.235 to 0.674]	0.537 [0.288 to 0.786]	0.499 [0.350 to 0.652]

The inter-observer reproducibility is indicated by kappa (κ) (and confidence interval) for different anatomic regions. NA indicates categories where the sign was not found at all and therefore the kappa could not be calculated. κ > 0.80 is excellent; 0.61 < κ < 0.80 is good; 0.60 < κ < 0.41 is moderate; κ < 0.40 is average. LN, lymph nodes; R, right; L, left.

**Table 3:** Intra-observer reproducibility of binary interpretations (negative/benign/non-significant or positive/malignant/high-risk

**Locations**	**Intra-observer agreement between first and second interpretation**			
	**kappa [confidence interval]**			
	**Senior 1 (CG)**	**Senior 2 (CC)**	**Junior 1 (JD)**	**Junior 2 (CP)**
Prostate and bed	0.493 [0.237 to 0.749]	0.789 [0.629 to 0.949]	0.798 [0.645 to 0.951]	0.534 [0.312 to 0.755]
Prostatectomy bed	NA	NA	NA	NA
Prostate	0.528 [0.252 to 0.805]	0.741 [0.561 to 0.920]	0.755 [0.585 to 0.925]	0.548 [0.320 to 0.776]
Pelvic LN	0.812 [0.656 to 0.967]	1.000 [1.000 to 1.000]	0.925 [0.823 to 1.000]	0.889 [0.766 to 1.000]
R hemi-pelvic LN	0.715 [0.485 to 0.944]	0.932 [0.799 to 1.000]	0.777 [0.569 to 0.896]	0.839 [0.662 to 1.000]
L hemi-pelvic LN	0.658 [0.380 to 0.935]	0.795 [0.571 to 1.000]	0.870 [0.693 to 1.000]	0.640[0.376 to 0.904]
PET overall conclusion	0.626 [0.432 to 0.820]	0.741 [0.561 to 0.920]	0.574 [0.368 to 0.780]	0.645 [0.421 to 0.869]

The inter-observer reproducibility is indicated by kappa (κ) (and confidence interval) for different anatomic regions. κ > 0.80 is excellent; 0.61 < κ < 0.80 is good; 0.60 < κ < 0.41 is moderate; κ < 0.40 is average. LN, lymph nodes; R, right; L, left.

Moreover, intra-observer reproducibility would certainly have been better if we had had access to each patient's clinical data. However, the aim of our study was to study the reproducibility of interpretation of foci using the [^18^F]choline PET/CT examination with different physicians, and not to validate the medical interpretation as such.

In our study, we did not have a Gold standard (prostatic or lymphatic biopsies). In our series, for some patients (22 of them), salvage treatment was radiotherapy or hormone therapy which did not give us access to anatomopathological results. A lumbo-aortic lymphatic salvage dissection was carried out for only three patients. For these three patients, the lymph nodes were considered suspicious by all the four physicians and this was confirmed by the anatomopathological result. Therefore, we cannot provide figures for sensitivity and specificity. However, the objective of our study was to answer the following question: Is there a focus of increased uptake indicating suspected recurrence? Our results show that concordance for a negative examination was excellent. It must be improved to confirm the reality of recurrence, particularly in cases where the prostate is in place; the results being better for lymphatic areas or the prostatectomy bed.

## Conclusion

The reproducibility of interpretation of imaging examinations is important because these examinations lead to decisions on the treatment to be given to patients. To our knowledge, our study is the first to report reproducibility of interpretation of [^18^F]choline PET/CT examinations. This study carried out on patients with prostate cancer in biochemical recurrence shows that physicians are in agreement in their interpretation of sub-diaphragmatic lymphatic recurrences. On the other hand, interpretation of the [^18^F]choline PET/CT examination is not so useful at prostate level in patients not previously treated with prostatectomy, but is of great interest in patients treated by prostatectomy. This is probably an argument in favour of systematic dual interpretation. Other reproducibility studies taking into account standardized uptake values (SUVs) are needed.

## Competing interests

The authors declare that they have no competing interests.

## Authors' contributions

CP is the first author. She coordinated all actions around this article. More specifically, she defined the protocol of this study, analysed the 60 examinations included in this study twice, and wrote the manuscript. CGC is the second author; she analysed the 60 examinations included in this study twice, supported the writing of the manuscript, and took part of its final review and validation. JG performed the statistical calculations. JD analysed the 60 examinations included in this study twice. CC analysed the 60 examinations included in this study twice and took part to the final review and validation of the manuscript. CL and MDD brought their deep knowledge about the choline tracer and supported the literature review. AC, JR, and DF contributed great advices, specifically on the [^18^F]choline PET/CT examination and for their daily support during this study. HB is the next to last author; he took part to the final review and validation of the manuscript. JPV is the last author; he had the original idea of this study, supported the writing of the protocol and the manuscript, and took part to the final review of it. All authors read and approved the final manuscript.
